# Wound Myiasis Complicating a Malignant Wound Caused by Merkel Cell Carcinoma of the Upper Lip: A Case Report

**DOI:** 10.7759/cureus.115925

**Published:** 2026-09-07

**Authors:** Ryo Nakanishi, Kazutoshi Nishimura, Yuto Yamamura, Atsushi Otsuka

**Affiliations:** 1 Department of Dermatology, Kindai University Hospital, Osaka, JPN

**Keywords:** elderly, malignant wound, merkel cell carcinoma, wound care, wound myiasis

## Abstract

Wound myiasis is a relatively rare condition characterized by the infestation of an open wound with fly larvae and may complicate malignant wounds associated with locally advanced cancers. We report a case of wound myiasis complicating a malignant wound caused by locally advanced Merkel cell carcinoma (MCC) of the upper lip. An octogenarian man with MCC of the left upper lip (cT2N1M0, Stage III) developed local recurrence after radiotherapy and was subsequently treated with avelumab followed by paclitaxel. Five days after the second cycle of paclitaxel, he presented with a chief complaint of tumor malodor. Physical examination revealed approximately 20 fly larvae within the ulcerated and necrotic tumor, leading to a diagnosis of wound myiasis. The patient was treated with saline irrigation and mechanical removal of the larvae and necrotic tissue. Thereafter, daily wound care was continued, and no recurrence of myiasis was observed.

In this patient, advanced age, impaired physical function, and interruption of wound care because of pain were considered to have contributed to the development of wound myiasis. Because wound myiasis is a potentially preventable complication, appropriate pain control, continuous management of malignant wounds, and the establishment of a multidisciplinary support system involving family members and caregivers are essential in elderly patients with skin cancer.

## Introduction

With the progressive aging of the Japanese population, an increasing proportion of patients with skin cancer are older adults. A nationwide epidemiological analysis based on the data from the Japanese National Cancer Registry demonstrated that most patients with skin cancer are elderly, with the age at diagnosis predominantly clustering in the seventh and eighth decades of life [1]. Elderly patients often present with locally advanced disease because of impaired physical or cognitive function, multiple comorbidities, or adverse social circumstances such as living alone or having insufficient caregiver support. Consequently, managing malignant wounds characterized by ulceration, bleeding, exudate, and malodor has become an important challenge for maintaining patients' quality of life (QOL).

Merkel cell carcinoma (MCC) is a rare and highly aggressive neuroendocrine skin cancer. Locally advanced MCC may develop ulceration, necrosis, bleeding, and malodor, resulting in the formation of a malignant wound. Wound myiasis, defined as the infestation of open wounds by dipterous fly larvae, is a rare but serious complication of such malignant wounds. Necrotic tissue, bleeding, and malodorous wounds provide a favorable environment for larval infestation [2,3]. Although wound myiasis associated with cutaneous malignancies has been reported, cases complicating MCC remain exceedingly rare. We report a case of wound myiasis arising from an ulcerated, locally advanced MCC of the upper lip. We also discuss the clinical importance of malignant wound management in elderly patients with skin cancer and review the relevant literature.

## Case presentation

An octogenarian man presented to our department with a rapidly enlarging mass on the left upper lip that had developed over the previous month. His activities of daily living were limited; he ambulated with a cane indoors and used a wheelchair outdoors. His Eastern Cooperative Oncology Group (ECOG) performance status was 1. Physical examination revealed a violaceous nodule measuring 22 × 18 mm on the left upper lip, accompanied by left submandibular lymphadenopathy (Figure 1A).

**Figure 1:**
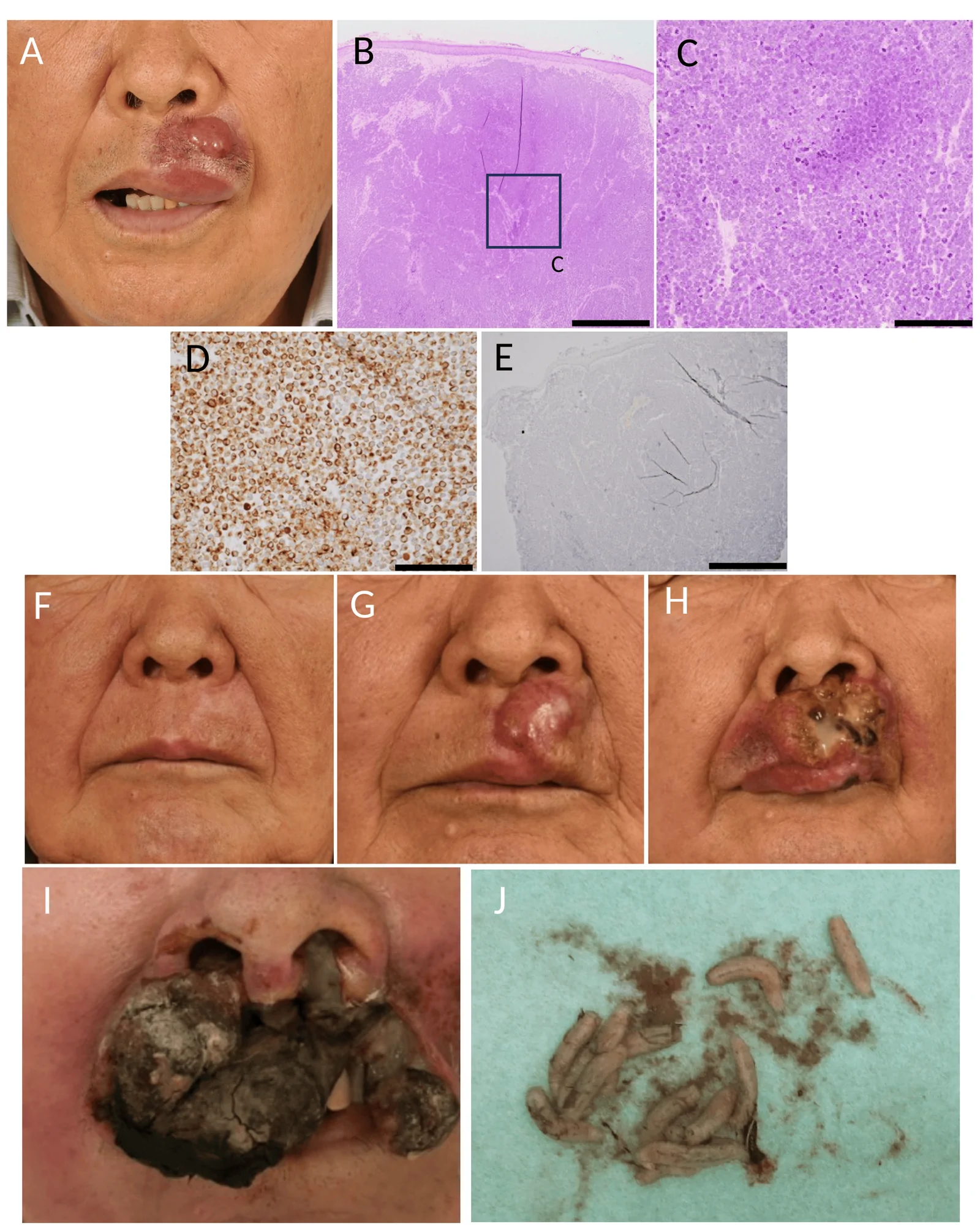
Clinical and histopathological findings (A) Initial presentation showing a rapidly growing 22 × 18 mm violaceous nodule on the left upper lip. (B) Low-power histopathological view demonstrating a dermal tumor nodule without epidermal connection. (C) High-power view showing proliferation of small basophilic cells with round nuclei. (D) Tumor cells positive for cytokeratin 20 (CK20). (E) Tumor cells negative for thyroid transcription factor-1 (TTF-1). (F) Partial tumor regression after radiotherapy. (G) Local recurrence six months after radiotherapy. (H) Progressive disease after four cycles of avelumab. (I) Ulcerated malignant wound complicated by wound myiasis. (J) Maggots removed from the ulcerated tumor. Scale bars: 250 μm (B, E); 100 μm (C, D)

Histopathological examination demonstrated a dermal tumor nodule with no continuity with the overlying epidermis on low-power magnification. High-power examination revealed a proliferation of small basophilic cells with round nuclei (Figures 1B, 1C). Immunohistochemically, the tumor cells were positive for cytokeratin 20 (CK20) and negative for thyroid transcription factor-1 (TTF-1), confirming the diagnosis of MCC (cT2N1M0, stage III according to the eighth edition of the American Joint Committee on Cancer (AJCC) staging system) (Figures 1D, 1E).

Considering the patient's advanced age and the anticipated functional and cosmetic impairment associated with upper lip resection, definitive radiotherapy (55 Gy in 22 fractions) targeting both the primary tumor and regional lymph nodes was selected over surgical excision. Although the tumor initially regressed following radiotherapy (Figure 1F), local recurrence developed approximately six months later (Figure 1G). The recurrent lesion was treated with four cycles of avelumab; however, progressive disease was observed (Figure 1H). Given the patient's general condition, treatment was subsequently switched to dose-reduced paclitaxel at 70 mg/m².

Five days after the second cycle of paclitaxel, the patient returned to our outpatient clinic with a complaint of a foul odor emanating from the tumor (Figure 1I). His body temperature was 36.5°C. Laboratory examination showed a white blood cell count of 6,600/μL, an absolute neutrophil count of 3,993/μL, and a C-reactive protein level of 0.7 mg/dL. He had no fever, cellulitis, purulent discharge, or other clinical evidence of secondary bacterial infection. Detailed history-taking revealed that he had been unable to adequately cleanse the wound for several days because of severe pain.

Irrigation with normal saline and partial debridement of the necrotic tissue exposed approximately 20 live larvae within the ulcerated tumor (Figure 1J). All visible larvae were removed during a single procedure by saline irrigation and mechanical removal along with the necrotic tissue, without local anesthesia or additional analgesia. Careful exploration of the wound at the end of the procedure confirmed that no visible larvae remained. Based on these findings, the patient was diagnosed with wound myiasis complicating a malignant wound caused by MCC. Morphological and molecular identification of the larvae was not performed. The wound was examined daily thereafter, and no residual or recurrent larvae were identified.

Following hospitalization, daily wound irrigation was continued, and no recurrence of myiasis was observed. However, the local tumor continued to progress despite prior treatments with radiotherapy, avelumab, and paclitaxel and was complicated by recurrent bleeding. As further effective treatment options were limited, palliative wound care, including topical Mohs paste and topical metronidazole, was initiated. The patient subsequently died of progressive MCC.

## Discussion

Myiasis is an infestation caused by the larvae of flies belonging to the order Diptera, affecting humans and other vertebrates. Based on the anatomical site of infestation, myiasis is classified into cutaneous, wound, and cavitary forms [2,4]. The present case was classified as wound myiasis because fly larvae infested a malignant wound caused by locally advanced MCC. Wound myiasis typically develops in open wounds containing necrotic tissue, blood, exudate, and malodor, all of which strongly attract flies [2]. In malignant wounds, volatile organic compounds released from necrotic tumor tissue are also thought to contribute to fly attraction [5].

In a systematic review of 123 cases of myiasis associated with cutaneous malignancies, Cuestas et al. reported that squamous cell carcinoma was the most common underlying malignancy and that the head and neck were the most frequently affected anatomical sites. Pain exacerbation, malodor, necrosis, and bleeding were the most common clinical manifestations [3]. Furthermore, Gonçalves et al. reviewed 56 cases of myiasis associated with head and neck malignancies and found that most cases occurred in patients with squamous cell carcinoma or basal cell carcinoma, predominantly involving the periocular region, scalp, auricle, and oral cavity [5]. Consistent with these findings, individual case reports have also described wound myiasis complicating advanced cutaneous squamous cell carcinoma of the scalp and face, highlighting that wound myiasis can occur even in developed countries when advanced cutaneous malignancies are accompanied by ulceration and inadequate wound care [6,7]. However, to the best of our knowledge, only a few cases of wound myiasis complicating MCC have been reported, suggesting that the present case represents an extremely rare clinical entity.

In the present case, several factors likely contributed to the development of wound myiasis. In addition to the patient's advanced age and impaired physical function, the locally advanced MCC had ulcerated, resulting in a malignant wound characterized by necrotic tissue, exudate, and malodor. Furthermore, the patient's inability to adequately cleanse the wound for several days because of severe pain may have facilitated larval infestation. Established risk factors for wound myiasis include advanced age, impaired physical function, underlying medical conditions such as psychiatric or neurological disorders and diabetes mellitus, poor hygiene, and unfavorable socioeconomic conditions [2,3,8]. Previous reports involving patients with head and neck malignancies have similarly emphasized that delayed wound care, poor hygiene, and impaired self-care capacity are major predisposing factors for wound myiasis [5,9]. Therefore, clinicians should be aware that even a short interruption in wound care may predispose elderly patients with locally advanced cutaneous malignancies to wound myiasis.

The diagnosis of wound myiasis is generally straightforward because larvae can be identified by direct visual inspection. In contrast, identification of the causative fly species is valuable for elucidating the route of infestation, understanding local epidemiology, and establishing preventive strategies against recurrence [2]. In recent years, molecular identification using cytochrome c oxidase subunit I (COI) and 16S ribosomal RNA (16S rRNA) gene sequencing has complemented conventional morphological identification [10]. Accurate species identification is ideally achieved by combining morphological examination, including evaluation of the cephalopharyngeal skeleton and posterior spiracles, with molecular techniques such as COI and 16S rRNA sequencing [11]. In the present case, species identification could not be performed because the larvae had not been preserved. This represents a limitation of our report.

The cornerstone of treatment for wound myiasis consists of mechanical removal of all larvae followed by thorough wound irrigation. Additional debridement of necrotic tissue and systemic antibiotic therapy may be required in the presence of secondary bacterial infection [2,5]. Although several reports have described the use of antiparasitic agents such as ivermectin, no comparative studies have established their efficacy, and these agents are generally regarded as adjunctive rather than standard therapy [3,12]. In patients receiving palliative care for advanced malignancies, prompt larval removal combined with meticulous wound care and symptom control remains the mainstay of management, whereas antiparasitic agents are reserved for selected cases [13]. In the present case, wound irrigation with normal saline, together with removal of the larvae and necrotic tissue, followed by ongoing wound care, successfully prevented recurrence of wound myiasis.

In contrast, maggot debridement therapy (MDT), which utilizes sterile larvae of Lucilia sericata reared under controlled laboratory conditions, has been established as an effective method of debridement for chronic wounds. Mumcuoglu et al. reported complete debridement in 38 of 43 wounds (88.4%) treated with MDT [14]. However, MDT should be clearly distinguished from naturally occurring wound myiasis. Unlike the sterile larvae used in MDT, larvae causing spontaneous wound myiasis are not sterile and may introduce pathogenic microorganisms or cause additional tissue damage. Therefore, prompt removal of the larvae is essential in naturally acquired wound myiasis [2,3,12].

Prevention of wound myiasis requires meticulous wound care, including regular wound cleansing, appropriate wound dressings, control of malodor and exudate, and measures to prevent fly exposure [12]. When patients are unable to perform adequate self-care, early establishment of a multidisciplinary support system involving family members, caregivers, and visiting nurses, together with appropriate pain management, is essential to maintain wound hygiene and reduce the risk of infestation. This multidisciplinary approach is particularly important in elderly patients with locally advanced cutaneous malignancies, in whom deterioration of performance status and self-care ability frequently complicate long-term wound management [5,13].

The present case highlights that wound myiasis is a potentially preventable complication of malignant wounds in patients with locally advanced cutaneous malignancies, a clinical scenario that may become increasingly common in aging societies. Adequate pain control, appropriate wound dressings, regular wound cleansing, and education of family members and caregivers regarding wound management are important not only for maintaining patients' quality of life but also for preventing wound myiasis.

## Conclusions

We reported a rare case of wound myiasis complicating a malignant wound caused by locally advanced MCC of the upper lip. Advanced age, impaired physical function, and an interruption in routine wound care due to pain associated with the malignant wound were considered to have contributed to the development of myiasis. Because wound myiasis is a potentially preventable complication, appropriate pain control, continuous wound care, and the establishment of a multidisciplinary support system involving family members and caregivers are essential for prevention.
